# The Effect of Gel Microstructure on Simulated Gastric Digestion of Protein Gels

**DOI:** 10.1007/s11483-018-9518-7

**Published:** 2018-03-05

**Authors:** Mauricio Opazo-Navarrete, Marte D. Altenburg, Remko M. Boom, Anja E. M. Janssen

**Affiliations:** Food Process Engineering Group, Wageningen University, P.O. Box 17, 6700 AA Wageningen, The Netherlands

**Keywords:** Simulated gastric digestion, Plant proteins, Soy protein isolate, Pea protein concentrate, Protein gel structure

## Abstract

The objective of this study was to analyse the impact of the gel structure obtained by different heat-induced temperatures on the in vitro gastric digestibility at pH 2. To achieve this, gels were prepared from soy protein, pea protein, albumin from chicken egg white and whey protein isolate at varying temperatures (90, 120 and 140 °C) for 30 min. Gels were characterised prior to digestion via microstructure and SDS-PAGE analysis. Subsequently, the gastric digestion process was followed via the protein hydrolysis and HPSEC analysis up to 180 min. Peptides of different sizes (<5 kDa) were gradually formed during the digestion. Our results showed that gels induced at 140 °C were digested faster. The protein source and gelation temperature had great influence on the in vitro gastric protein digestibility.

**Electronic supplementary material:**

The online version of this article (10.1007/s11483-018-9518-7) contains supplementary material, which is available to authorized users.

## Introduction

Protein gels are widely used to provide structure in foods. Several proteins have the ability to form gels on heating with different structures, depending on the source and gelling conditions [1–3]. Protein gels can be prepared by cross-linking flexible proteins (e.g. gelatine and keratin) and by using protein aggregates of low-structured proteins (e.g. casein) or globular proteins (e.g. ovalbumin, whey proteins and soy proteins) [4].

Gelation often involves several reactions such as denaturation, dissociation-association, and aggregation. The kinetics of the reactions involved largely determine the type of structure formed [5]. The denaturation unfolds a native protein such that functional groups (such as sulfhydryl groups or hydrophobic groups) become exposed. These exposed groups may then interact to form aggregates. When the protein concentration is high enough, aggregation leads to the formation of a gel. At lower concentrations, the aggregation leads to precipitation of isolated protein aggregates [4, 6]. Protein gelation changes their rate of digestion [7]. Understanding this mechanism is important for the development of foods that control the rate of release of macronutrients and slow the rate of the stomach emptying, thus limiting the consumed amount of food [8]. Generally, plant proteins are less digestible than animal proteins [9], and the digestibility of their gels is probably also less than those of animal origin; however the gel structure will influence this as well. Soy and pea proteins are important food proteins in many-based food formulations [10, 11]. In soy, the main proteins are glycinin and β-conglycinin. Glycinin, having a molecular mass of 180 kDa, denatures at around 90 °C at neutral pH, while β-conglycinin, with a molecular weight between 150 and 200 kDa, denatures at 70 °C [4]. Pea protein consists of 90% of the globulins legumin, vicilin and convicilin and for 10% of the albumins PA1 and PA2 (Nutralys pea protein technical bulletin). The molecular weight of the globulins varies from 175 kDa for vicilin to 385 kDa for legumin (Nutralys pea protein technical bulletin), while the proteins denature around 85 °C [12].

During the gelation of proteins, a three-dimensional network of polypeptides, that is able to enclose water, is formed. There are two different classes of proteins gels: cross-linked protein networks and globular protein gel. The cross-linked protein networks are formed by flexible proteins being partially denatured. On the other hand, the globular proteins during unfolding exposes hydrophobic parts, which are situated in the middle of the protein before unfolding, which tend to form clusters.

Studies on the effect of gel structure on the protein digestibility of plant proteins are limited. The structure of soy protein gelled with different coagulants strongly influenced the protein bioaccessibility [13]. Bornhorst [14] indicated that hardness is an important predictor of food disintegration during gastric digestion: semi-soft or soft foods disintegrate faster than solid foods; liquid foods pass quickly through the stomach whereas solid foods remain in the stomach for longer times [15]. However, its relation to the digestion rate was not addressed.

The aim of this study is to investigate the impact of the protein source and microstructure obtained by different heat-induced temperatures on the in vitro gastric digestibility in a simulated gastric environment.

## Materials and Methods

### Materials

Soy protein isolate (SUPRO® 500E IP) (SPI) with a protein content of 83.4% (w/dw) was purchased from Solae (St. Louis, Missouri, USA). Pea protein concentrate (NUTRALYS® F85G) (PPC) was acquired from Roquette (France) with a protein content of 75% (w/dw). Whey protein isolate (WPI) (Bipro, lot no. JE 034–70–440-3) was supplied by Davisco Food International, Inc. (Le Sueur, USA) with a protein content of 99.3% (w/dw), while casein from bovine milk was supplied by FrieslandCampina (Wageningen, The Netherlands) with a protein content of 95.9% (w/dw). Albumin from chicken egg white (grade II) was purchased from Sigma-Aldrich (St. Louis, Missouri, USA) with a protein content of 92% (w/dw). The protein content of the sources was measured by Dumas analysis (Nitrogen analyser, FlashEA 1112 series, Thermo Scientific, Interscience, Breda, The Netherlands) in triplicate, using conversion factors of 5.71 for soy, 5.52 for pea, 6.25 for whey, 6.35 for casein and 6.45 for albumin from chicken egg white. Pepsin from porcine gastric mucosa (400–800 units/mg, P7125), mucin from the porcine stomach (Type III, M2378-100G) and all other chemicals were purchased from Sigma-Aldrich, Inc. (St. Louis, MO, U.S.A.). Milli-Q water (18.2 MΩ cm at 25 °C, Millipore Corporation, Molsheim, France) was used for all experiments.

### Preparation of Gels

#### Soy and Pea Protein Gels

SPI and PPC protein dispersions were prepared by suspending SPI and PPC powder in Milli-Q water (20 g protein/100 g) and mixed with a spatula until it was completely wet. Subsequently, the mixture was left standing for 3 h at room temperature, to ensure further dissolution. Later, the mixture was put into PTFE tube (inner diameter 1 cm and length 10 cm) with screw caps on both sides and then sealed. The tubes were rotated at 30 rpm and heated at 90 °C in a water bath for 30 min, while for the treatment at 120 and 140 °C heating was done in a glycerol bath for 30 min. Subsequently, the tubes were immediately placed in ice water and stored overnight in the fridge (4 °C). The next day the gels were carefully removed and analysed. The high temperatures were chosen considering some studies done with the same SPI source [16, 17]. While 90 °C was chosen considering the previous study about digestion of protein WPI and albumin from chicken egg white gels [18].

#### Albumin Gel from Chicken Egg White

Albumin protein gel was prepared by mixing of albumin powder in Milli-Q water (20 g protein/100 g) and stirred at room temperature for 3 h until that was completely dissolved. The solution was covered with a Parafilm (Pechiney Plastic Packaging, Inc., IL, U.S.A.) to prevent evaporation during stirring. After dissolution, the tubes were put in a water bath at 90 °C and rotated at 30 rpm for 30 min. For the heating at 120 and 140 °C, the heating was done in a glycerol bath for 30 min. After heating, the tubes were immediately placed in ice water and stored overnight in the fridge (4 °C). The next day the gels were carefully removed and analysed.

#### Whey Protein Gel

WPI powder was mixed with Milli-Q water (20 g protein/100 g) and stirred at room temperature for 3 h with a magnetic stirrer until the protein was completely dissolved. To prevent water evaporation, the solution was covered with Parafilm (Pechiney Plastic Packaging, Inc., IL, U.S.A.). After mixing, the solution was centrifuged (Thermo Scientific, MA, USA) at 3000 rpm for 20 min at 20 °C to remove air bubbles. Subsequently, the solution was put into the PTFE tube and heated the tubes were put in a water bath at 90 °C and rotated at 30 rpm for 30 min, while for the treatment at 120 and 140 °C heating was done in a glycerol bath for 30 min. After heating, the tubes were immediately placed in ice water and stored overnight in the fridge (4 °C). The next day the gels were carefully removed and analysed.

#### Scanning Electron Microscopy (SEM)

The gels were first dehydrated. Pieces were cut (1 × 1 × 0.5 cm) and fixed with 2.5 mL/100 mL glutaraldehyde in 0.1 mol/L phosphate buffer (pH 7.3) at room temperature. The samples were then rinsed with 0.1 mol/L phosphate buffer (pH 7.3) and dehydrated in a substitution series of 50, 70, 80, and 90 mL/100 mL ethanol, for 15 min in each solution followed by three times for 30 min in absolute ethanol. The samples were vacuum dried at room temperature and mounted in carbon tabs (SPI Supplies/Structure Probe Inc., West Chester, USA) to fix the samples on aluminium pin mounts (SPI Supplies/Structure Probe Inc., West Chester, USA) for SEM examination (Phenom G2 Pure, Phenom-World BV, Eindhoven, The Netherlands).

#### Texture Analysis

Gels were cut into cylinders of 1 cm diameter and 1 cm height. Uniaxial single compression tests were performed at room temperature using a texture analyser with a 100 N load cell (type 5564, Instron, MA, USA) equipped with a 50 mm cylindrical probe. The probe travelled to 5 mm distance to the tray at a speed of 5 mm/min. During the test run, the resistance of the sample was recorded for every 0.01 s and plotted as the absolute force (N) versus time (s). The gel hardness was defined as the maximum peak force attained during the compression. Five cylinders were measured for each protein gel type.

#### SDS–Page

The molecular characterisation of the gels was done by reducing SDS polyacrylamide gel electrophoresis. Before electrophoresis, the protein gels were cut into small pieces. The samples were then diluted with sample buffer (0.5 M Tris–HCl, pH 6.8; 2% *v*/v SDS; 2.5% v/v glycerol; 0.2% v/v bromophenol blue; 0.5% v/v 2-mercaptoethanol). The weight ratio of sample-to-buffer was 1:1. Each sample was heated to 90 °C for 4 min in an Eppendorf thermomixer (Eppendorf AG, Hamburg, Germany). The samples were then centrifuged at 10,000 *g* for 5 min. An amount of 12 μL of each sample and molecular weight markers Precision Plus Protein All Blue Standards (Bio-Rad Laboratories Inc., Hercules, USA) were loaded on a 12% Tris–HCl Mini-PROTEAN TGX Precast Gel (Bio-Rad Laboratories Inc., USA). The electrophoresis was carried out at 200 V for about 1 h. Afterwards, the gel was stained with Bio-safe Coomassie Stain (Bio-Rad Laboratories Inc., USA) and gel images were taken using a GS-900 Calibrated Densitometry System (Bio-Rad Laboratories, Inc., USA).

#### Preparation of Protein Solutions

Solutions were prepared by dissolving a mass equivalent to 0.1 g of protein from all different protein sources into 2 mL Eppendorf tube with Milli-Q water. The protein mixtures were stirred at room temperature for 30 min at room temperature and used for gastric digestion.

#### In Vitro Gastric Digestion of Protein Gels and Solutions

Simulated gastric juice (SGJ) was prepared according to Avila [19] with some modifications. Pepsin (1 g/L) and mucin (1.5 g/L) were dissolved in Milli-Q water and the pH was adjusted to 2.0 with HCl. Additionally, some experiments were performed using NaCl (8.775 g/L) to study the effect of salt on the enzyme activity. The simulated gastric digestion experiments were performed with 50 mL SGJ in a jacketed glass vessel connected to a water thermostat bath at 37 °C (Julabo GmbH, Seelbach, Germany) for 3 h. Stirring was done at 100 rpm and the vessel was sealed with Parafilm (Pechiney Plastic Packaging, Inc., IL, U.S.A.) to avoid evaporation.

Based on the work of Jalabert-Molbes [20] on different kind of foods, cylindrical samples were cut (3 mm diameter × 3 mm height approximately) of each protein source with a puncher. Using these cylinders, a certain mass equivalent to 0.1 g of net protein was digested in 50 mL SGF, while for solution experiments, 0.1 g of protein in 2 mL Milli-Q water was digested in 50 mL SGF.

Samples were taken at 20, 60, 120 and 180 min for further analyses. Immediately after sampling, the samples were heated in a pre-heated Eppendorf thermomixer (Eppendorf AG, Hamburg, Germany) at 90 °C and 1400 rpm for 5 min to inactivate the pepsin, which is rapidly inactivated at a temperature above 62 °C [21]. All digestion experiments were done in triplicate.

#### Size Exclusion Chromatography (HPSEC)

The composition of the SGF during and after in vitro gastric digestion was analyzed via high-performance size-exclusion chromatography (HPSEC) using an Ultimate 3000 UHPLC system (Thermo Scientific, MA, U.S.A.) equipped with a TSKgel G3000SWxl column (7.8 mm × 300 mm) (Tosoh Bioscience LLC, King of Prussia, PA, U.S.A.) and TSKgel G2000SWxl (7.8 mm × 300 mm) (Tosoh Bioscience LLC, King of Prussia, PA, U.S.A.) connected in line. For this analysis, 10 μL of undiluted sample was used. The mobile phase was acetonitrile (30%) in Milli-Q water (70%) buffer containing trifluoroacetic acid (0.1%). The flow rate was 1.5 mL/min and the UV detector was set at 214 nm. Calibration was done with thyroglobulin (670 kDa), g-globulin (158 kDa), ovalbumin (44.3 kDa), α-lactalbumin (14.1 kDa), aprotinin (6.51 kDa), insulin (5.7 kDa), bacitracin (1.42 kDa) and phenylalanine (165 Da) (Sigma-Aldrich, Inc., St. Louis, MO, U.S.A.). The molecular mass was estimated based on the elution time of the molecular weights markers. All measurements were done in duplicate.

#### DH

The free amino groups (mM) were measured using the o-phthaldialdehyde (OPA) assay method in order to determine the degree of hydrolysis attained. The OPA reagent (100 mL) was prepared by dissolving 3.81 g sodium tetraborate decahydrate (Borax) and 0.1 g of SDS in 80 mL milli-Q water. 80 mg of o-phthaldialdehyde, that was dissolved in 2 mL ethanol, was then added to the Borax-SDS solution together with 88 mg of dithiothreitol (DTT). The solution was filled up to 100 mL with milli-Q water and filtered over a 0.45 μm filter. This solution was stored in a bottle covered with aluminium foil because the OPA reagent is sensitive to light.

A standard curve was prepared using L-serine in a concentration range of 50–200 mg/L. The OPA assay was carried out by the addition of 200 μL of sample (or standard) to 1.5 mL of OPA reagent. The absorbance of these solutions was measured after 3 min at 340 nm with a spectrophotometer DU 720 (Beckman Coulter Inc. Pasadena, CA, U.S.A). Free amino groups were expressed as serine amino equivalents (Serine NH_2_). The DH was calculated with the following equations:

1 $$ DH=\frac{h}{h_{tot}}\cdot 100\% $$

2 $$ h=\frac{Serine\;{NH}_2-\beta }{a} $$

Where *α*, *β*, and *h*_*tot*_ values reported by Adler-Nissen [22] are used here (Table 1). All measurements were done in triplicate.

**Table 1:** Value of constants *α*, *β* and *h*_*tot*_ for different protein sources [22]

Protein	*α*	*β*	*h*_*tot*_ (meqv/L)
Soy	0.970	0.342	7.8
Pea	1.00	0.40	7.4
Casein	1.039	0.383	8.2
Whey	1.00	0.40	8.8
Albumin	1.00	0.40	9.0

#### Statistical Analysis

Significance testing was performed using Fisher’s least significant difference (LSD) test, and the differences were taken to be statistically significant when the *p*-value was <0.05. The multiple range test (MRT) included in the statistical program was used to prove the existence of homogeneous groups within each of the parameters analysed. The analysis was performed using Statgraphics Centurion XVI Statistical Software (Statistical Graphics Corp., Herdon, USA).

## Results and Discussion

### Gel Characterization

#### SDS-PAGE Analysis of Gels

**Fig. 1:**
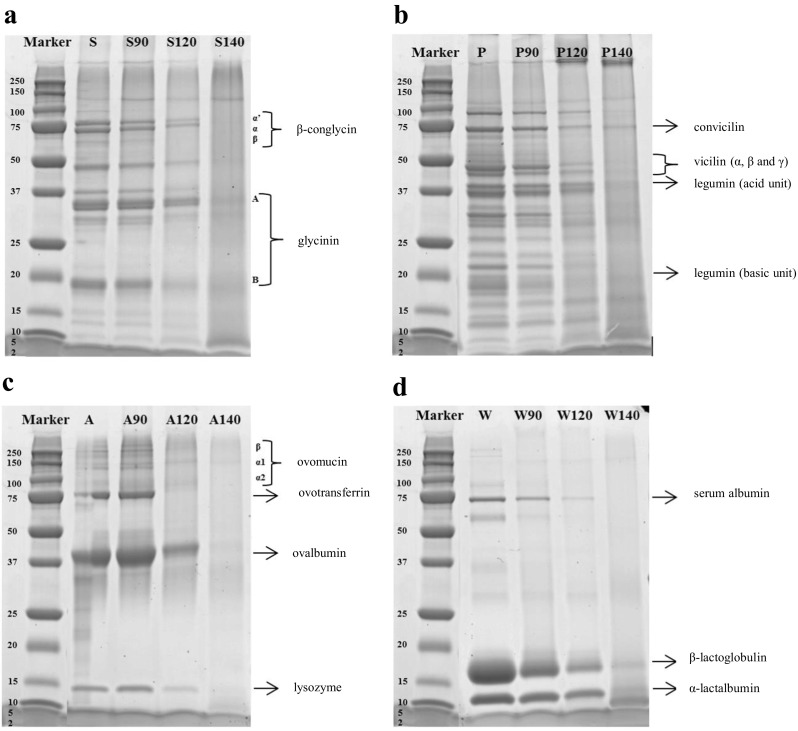
SDS-PAGE profiles of protein gels: (**a**) S (soy protein isolate), S90 (soy protein gel made at 90 °C), S120 (soy protein gel made at 120 °C), S140 (soy protein gel made at 140 °C), (**b**) P (pea protein concentrate), P90 (pea protein gel made at 90 °C), P120 (pea protein gel made at 120 °C), P140 (pea protein gel made at 140 °C), (**c**) A (albumin from chicken egg white) A90 (albumin from chicken egg white protein gel made at 90 °C), A120 (albumin from chicken egg white protein gel made at 120 °C), A140 (albumin from chicken egg white protein gel made at 140 °C) and (**d**) W (whey protein isolate), W90 (whey protein gel made at 90 °C), W120 (whey protein gel made at 120 °C), W140 (whey protein gel made at 140 °C)

As the electrophoretic analyses of the gels in Fig. 1 show, the protein subunits of the soy protein (7S–globulins and 11S–globulins) gelled at 90 °C did not show any change, while those gelled at 120 °C exhibited faint bands of glycinin. The 7S proteins (glycinin) could have formed large aggregates that were not able to penetrate the gel (Fig. 1a). SPI gels made at 140 °C did not yield any bands anymore because of large protein-protein complexes, possibly covalently cross-linked, were formed that most likely were not able to dissolve in the sample buffer.

The protein banding pattern of pea proteins (Fig. 1b) gelled at 90 °C were identical to the ungelled protein. Bands can be seen ranging from ~100 to ~10 kDa that originate mainly from legumin and vicilin, which are 11S and 7S globulins, respectively. Legumin, a hexameric protein, dissociates into two subunit peptides (α; acidic 38–40 kDa and β; basic 19–22 kDa) when the S–S bonds are broken under reducing conditions [7, 23]. Vicilin is a trimeric protein, composed of three heterogeneous subunits of ~50 and convicilin ~70 kDa. No S–S bonds are involved in the vicilin protein superstructure [7, 24]. Gels made at 120 °C still showed faint bands while gels made at 140 °C did not show any bands anymore. Both gels show some large protein-protein complexes, possibly covalently crosslinked, that were unable to penetrate the pores of the SDS PAGE gel.

Gels of animal proteins showed very similar behaviour. Albumin from chicken egg white (Fig. 1c) gelled at 120 °C show that the ovomucin and ovotransferrin proteins bands gradually disappeared, and for gels made at 140 °C all bands had disappeared. The most abundant proteins in WPI are β-lactoglobulin and α-lactalbumin (Fig. 1d). With the increase of the temperature, the change in the intensity of serum albumin, β-lactoglobulin and α-lactalbumin bands is shown. Also here, all bands were gone for gels made at 140 °C. There is no evidence that heating for 30 min at 140 °C or at the other temperatures could cause hydrolysis of peptide bonds (see supplementary material Fig. 1). To evaluate this, gels were ground and dissolved overnight in a solvent consisting of 8 mol/L urea and 0.03 mol/L dithiothreitol (DTT). The dissolved gels were then analyzed by HPSEC. The chromatograms showed that gels formed at different temperatures presented practically the same curves from elution time of 15 min, which is equivalent at a molecular weight (MW) of 153 Da. Therefore, the temperatures and heating time used do not cause hydrolysis of peptide bonds. However, after heating at 140 °C is evident the protein aggregation after protein denaturation when hydrogen bonds and other interactions that stabilize its tertiary structure, are weakened causing the protein to unfold and subunits to dissociate.

#### Gel Morphology

**Fig. 2:**
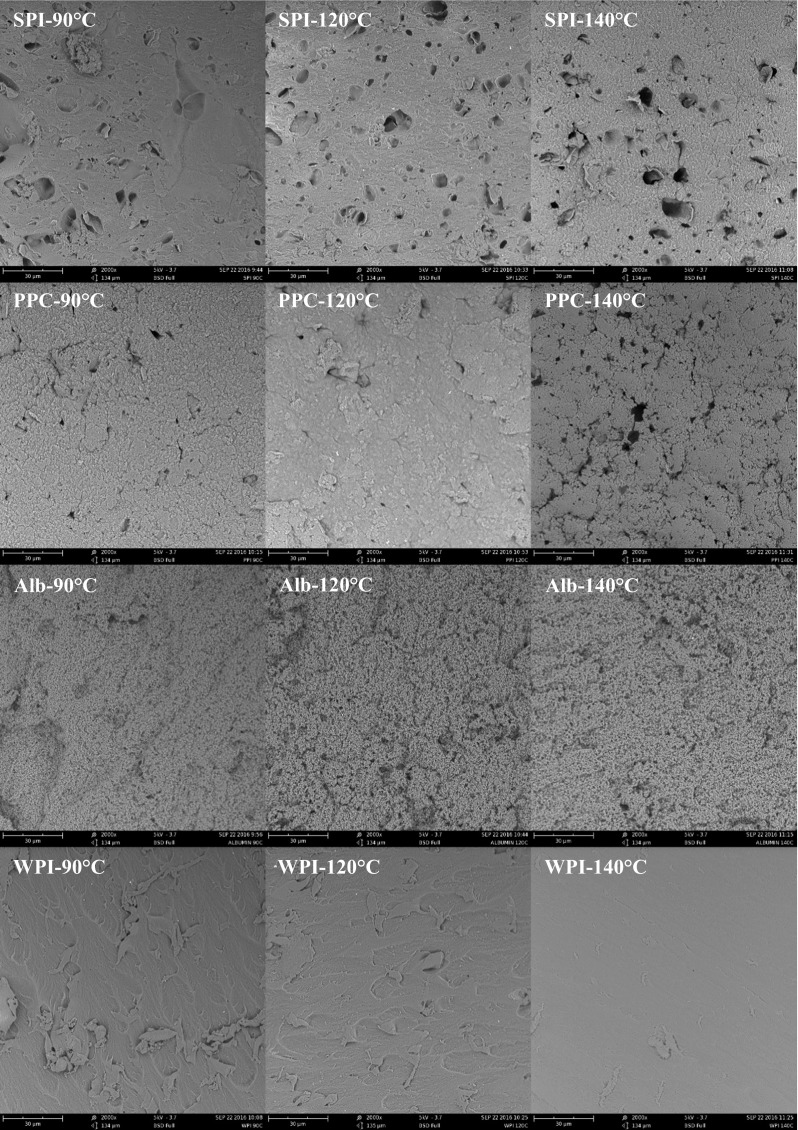
SEM images of protein gels made at different temperatures (90, 120 and 140 °C) using different sources. SPI, soy protein isolate; PPC, pea protein concentrate; Alb, albumin from chicken egg white; WPI, whey protein isolate

The microstructures of the four different protein types gelled at three different temperatures were examined using SEM (Fig. 2). For the SPI gels, not structure differences were observed between the different gelling temperatures. The PPC gelled at 140 °C seems to present a more fragile structure than the PPC gelled at 90 and 120 °C. This fragility might result in a fast gel breakdown and thus faster protein digestion. Proteins from animal origin sources yield different structures. While WPI gelled at different temperatures did not show any change in morphology, albumin from chicken egg white gelled at 90 °C showed a more compact structure in comparison to the gels made at 120 and 140 °C. This more compact structure might result in slower gel disintegration and therefore slower protein digestion.

**Fig. 3:**
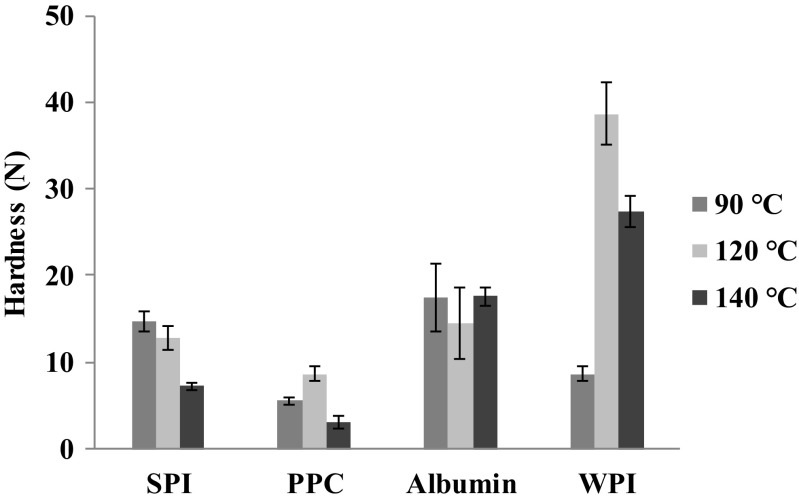
Hardness prior digestion of the different protein gels. SPI, soy protein isolate; PPC, pea protein concentrate; Albumin, albumin from chicken egg white; WPI, whey protein isolate

Texture analysis was performed by measuring hardness where it was related to the peak force of the compression cycle. The hardness (N), shown in Fig. 3, was different for all studied gels. WPI gelled at 90 and 120 °C presented significantly higher (*p* < 0.05) hardness values of 27.4 and 38.6 N, respectively, compared with to the other gels. In contrast, albumin from chicken egg white did not present significant differences (*p* > 0.05) with any gelling temperature. For both plant protein gels, SPI and PPC, gelling at 140 °C resulted in the weakest gel, which could result in faster gel disintegration during digestion.

As the physical integrity of gels depends on the balance between attractive and repulsive strengths of the protein molecules involved in the system [25]. If the attractive strengths predominate, a coagulum is formed, and water is driven off the network matrix. If the repulsive strengths dominate, a three-dimensional network can not be formed [26].

The establishment of gel networks at 85 to 90 °C is attributed to the formation of covalent linkages, to the changes of the thiol group to disulphide linkages, and to hydrophobic interactions [27]. These interactions between nonpolar segments of adjacent polypeptides occur only if these polypeptides are opened, induced by heating. Cooling increases the hydrogen bonds.

However, heating at high temperatures could also result in progressively lower protein solubility and therefore hydrogen bonding is weakened. Furukawa [28] in a study on soy protein gels found that the gel hardness increased with heating temperature up to 80 °C, but the weakening occurred at higher temperatures, especially those greater than 110 °C. Based on processing temperature, they classified the gel as soft (<50 °C), hard (60 to 110 °C), and fragile (>120 °C). This is in accordance with the results obtained on plant-based proteins in our research. The mechanism of gel formation was suggested to be cross-linking of soy proteins via disulfide and hydrogen bonding and hydrophobic interactions which controlled by temperature [28, 29]. However, the animal-based proteins (albumin and WPI) presented a different behaviour.

During heating, albumin is polymerized by intermolecular exchange linkages from sulphydrilic groups to disulphide linkages, which makes a network. Thermo-coagulation requires a balance of electrostatic attractions between protein molecules and hydrophobic interactions during the gel formation [30]. The intermolecular disulphide linkages increase the stability of the gel matrix. The increased size of polypeptide chains can delay the rupture of non-covalent interactions, and favour the gel network stability.

When whey protein solution is heated at a sufficiently high temperature (75 °C), the protein molecules unfold and interact to form intermediate aggregates prior to the formation of a gel network [31]. The formation of intermediate aggregates involves two broad types of bonding: covalent and non-covalent bonding. The former consists of inter and intramolecular disulphide bonds [32] formed via sulphydryl–disulphide interchange or sulphydryl oxidation reactions [33]. The latter are non-covalent interactions, such as hydrophobic, hydrogen bonding, ionic and other weak interactions that also contribute to the formation of aggregates and a gel network [34].

The non-covalent interactions, such as hydrophobic and ‘Van der Waals’ interactions, hydrogen bonds and ionic interactions, are related to the nature of the protein, to its concentration, to the solution pH, to the denaturation intensity caused by heating and by the ionic medium [35], and interfere with the attractive and repulsive strengths of the three-dimensional network. Differences in gel-forming ability among globular proteins generally reflect the variety of degrees of protein-protein interactions and the number and extension of interactive sites available within the opened molecule [27]. Therefore, the differences in the gel hardness could be simply related to the nature of the protein source.

#### Hydrolysis of Protein Gels

Since many foods and meals contain significant amounts of salts, and it is known that this influences the behaviour of protein gels, the effect of NaCl on the rate of hydrolysis was studied. To assess this effect, we used 5% SPI and PPC solutions in SGJ with and without NaCl. In fact, SPI and PPC solutions digested in SGJ with and without NaCl did not show significant differences (*p* > 0.05) in the rates of hydrolysis (see supplementary material Fig. 2). For this reason, further experiments were performed without NaCl added to the SGJ.

**Fig. 4:**
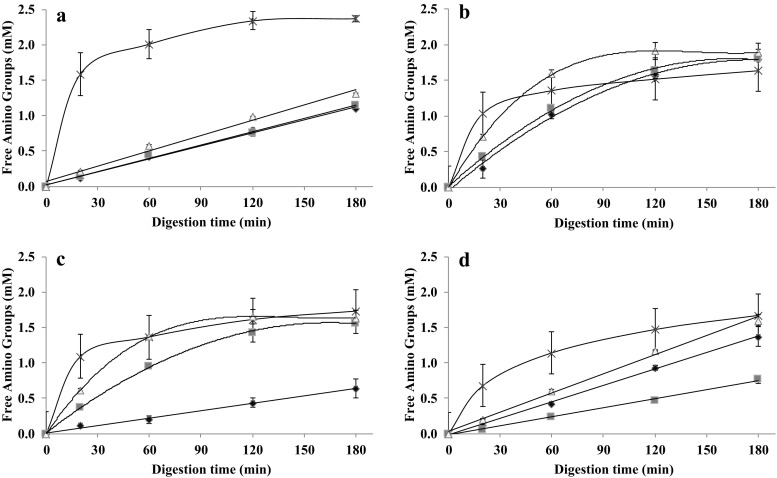
Hydrolysis profile of proteins solutions (×) and proteins gelled at 90 °C (◆), 120 °C (■) and 140 °C (△). **a** Soy protein gels, (**b**) pea protein gels, (**c**) albumin from chicken egg white gels and (**d**) whey protein gels

The in vitro gastric rate of hydrolysis of gels of SPI, PPC, albumin from chicken egg white and WPI was measured in time and is shown in Figs. 4a–d, respectively. The hydrolysis profile of PPC (Fig. 4b) and albumin from chicken egg white (Fig. 4c) made at 140 °C increased rapidly in the first 60 min of digestion by pepsin, and then approached a plateau from 60 to 180 min. The SPI and WPI gels hydrolysed very slowly, more or less constantly during the full 180 min of digestion.

The protein hydrolysis of the SPI gels (Fig. 4a) made at 140 °C was somewhat, but significantly higher (*p* < 0.05) than those made at lower temperatures. This may be related to their microstructure: the gel made at 140 °C appeared more porous (Fig. 2). Along with this, the lower hardness of the gels made at 140 °C (Fig. 3) is consistent with faster disintegration. Similar results were found with soft agar gel beads which disintegrated quickly in the human stomach whereas harder beads were broken down more slowly [36]. The same was observed with soft whey protein emulsion gels [37]. Our PPC gels presented a significantly higher (*p* < 0.05) protein hydrolysis after 180 min of digestion than the gels from other proteins (Fig. 4b). The PPC gels made at 140 °C presented the fastest initial protein hydrolysis, however, after 180 min of digestion, all PPC gels converged to the same hydrolysis values (*p* > 0.05). The PPC gels made at 140 °C showed a more fragile structure (Fig. 2), which is consistent with their low hardness (Fig. 3). We expect that these gels disintegrated quickly, and hence exposed a larger surface area for faster enzymatic initial hydrolysis. The final plateau DH value of around 7% is probably related to the type of peptide bonds available for hydrolysis.

The digestion of the albumin from chicken egg white gels made at 90 °C yielded a significantly slower (*p* < 0.05) hydrolysis during 180 min of digestion (Fig. 4c), while the gels made at 120 and 140 °C showed much faster initial hydrolysis followed by convergence towards a plateau DH value of around 5%. The SEM analysis (Fig. 2) showed that the gels made at 90 °C had a more compact microstructure than the gels made at higher temperatures. The lower disintegration rate would explain the much slower hydrolysis. In this case, the hardness (Fig. 3) is not correlated with the rate of hydrolysis. The hydrolysis of the WPI gels (Fig. 4d) all followed a linear trend, with the WPI gels made at 140 °C giving significantly higher protein hydrolysis values (p < 0.05) and DH value (around 5%). Also here, the microstructure analysis (Fig. 2) and hardness analysis (Fig. 4) does not correlate with the rate of hydrolysis of these gels.

#### Hydrolysis of Protein Solutions

The hydrolysis of the different protein isolates in solution was also followed (Fig. 4a–d). WPI in solution showed a significantly faster hydrolysis (p < 0.05) than casein; the hydrolysis of the casein in solution was slow but almost constant in time. The digestion rate is normally used to categorised into “slow” and “fast” digestibility, based on the time-dependent rise in plasma amino acids after food intake. The concept of slow and fast proteins, based on the rate at which blood plasma levels of amino acids rise, was first described by Boirie [38]. They indicated that after ingestion, the absorption peak of whey proteins occurs between 40 min and 2 h after ingestion, while the rise in plasma amino acids after casein intake continues for 7 h. This different hydrolysis behaviour is related to the coagulation that casein undergoes under acidic gastric conditions, forming a protein network resulting in a reduced accessibility to gastric digestive enzymes and thus delayed gastric emptying. Native whey proteins stay in solution at the same pH and thus remain fully accessible to the gastric digestive enzymes [39]. Thus, whey protein is a reference fast protein and casein a reference slow protein. The other sources (SPI, PPC and albumin from chicken egg white) presented even faster hydrolysis in solution than WPI, especially in the first 20 min, so these proteins have fast digestibility as well. Albumin from chicken egg white in solution stands out for its significantly highest digestibility (*p* < 0.05), which is in contrast to the rate of hydrolysis of its gels (Fig. 4c).

In our experiments, the final level of hydrolysis for the SPI in solution was much higher than the values attained with a gel, but the slow hydrolysis rate of the gel is indicative of very slow mass transfer. In contrast, the PPC in solution attained a final DH of around 6%, which is in the same range as obtained for the gel. We conclude that the PPC gels are more open and porous than the SPI gels, and therefore offer much better access for the enzyme to act upon the gel.

#### Size-Exclusion Chromatography (HPSEC) Analysis

**Fig. 5:**
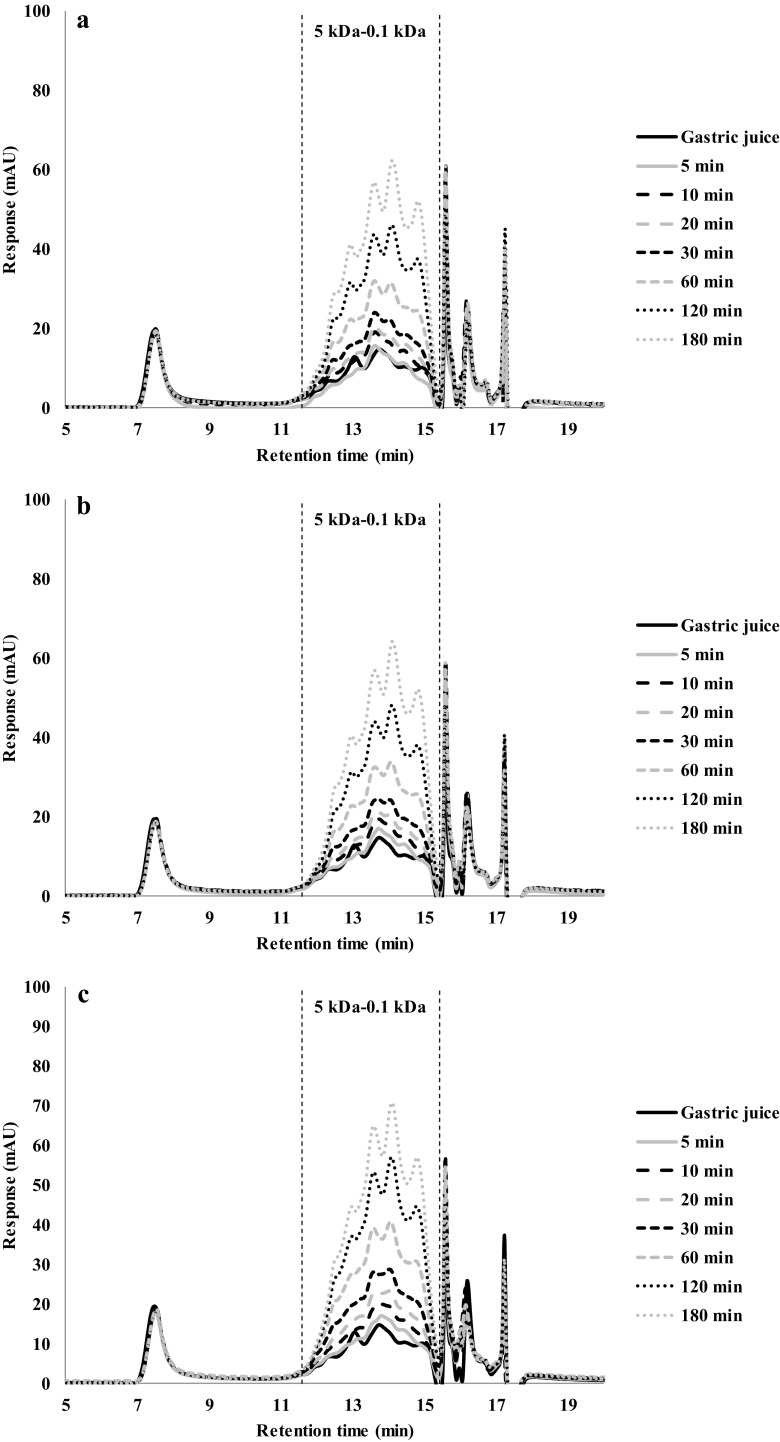
HPSEC profiles of gastric digestion of soy protein gels made at: (**a**) 90 °C, (**b**) 120 °C and (**c**) 140 °C

**Fig. 6:**
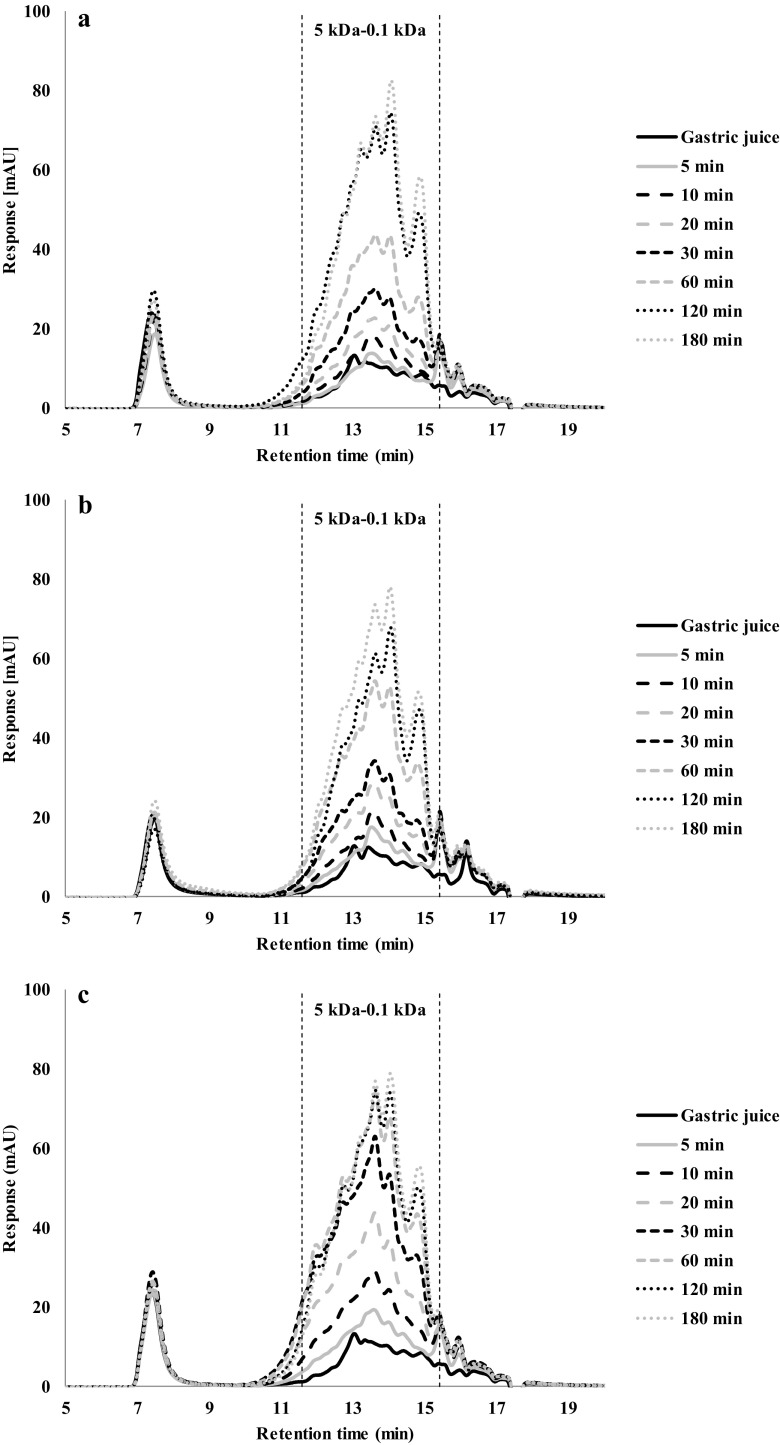
HPSEC profiles of gastric digestion of pea protein gels made at: (**a**) 90 °C, (**b**) 120 °C and (**c**) 140 °C

**Fig. 7:**
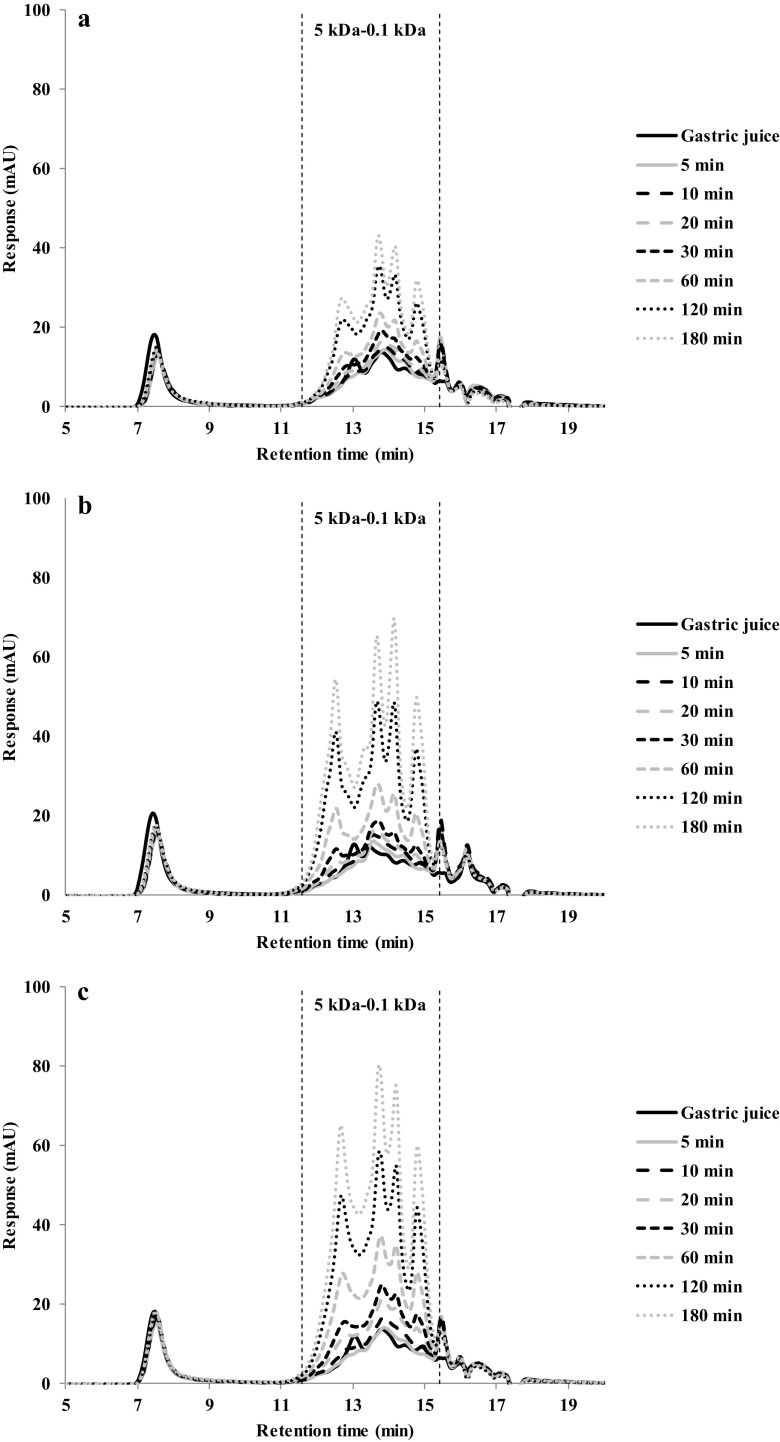
HPSEC profiles of gastric digestion of albumin from chicken egg white gels made at: (**a**) 90 °C, (**b**) 120 °C and (**c**) 140 °C

**Fig. 8:**
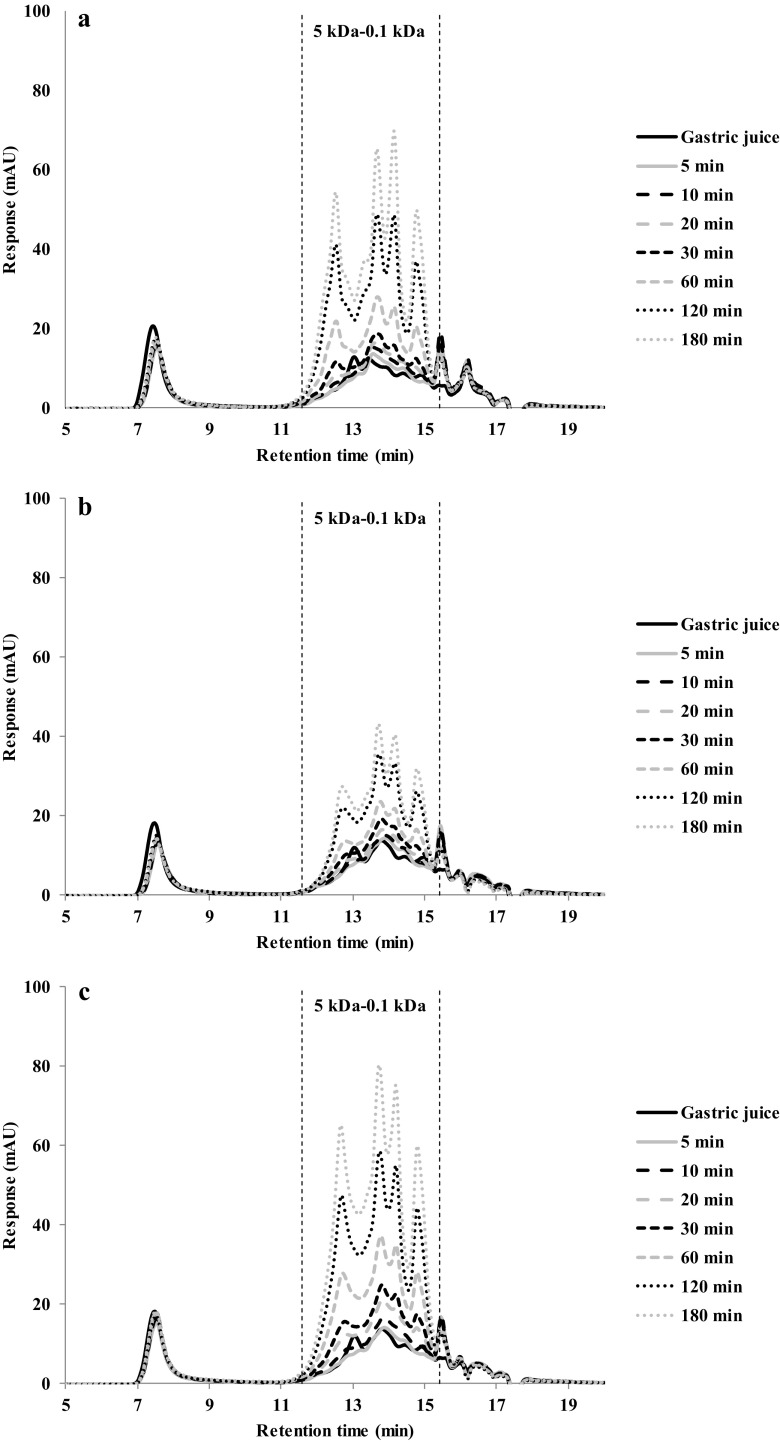
HPSEC profiles of gastric digestion of whey protein gels made at: (**a**) 90 °C, (**b**) 120 °C and (**c**) 140 °C

The simulated gastric fluid samples taken from the digestion of protein gels were analysed with HPSEC (Figs. 5, 6, 7 and 8). Typically, small peptides ranging from 5 to 0.1 kDa were released over time. There was no discernible difference between the chromatograms made with gels prepared at different temperatures (Fig. 5). This is consistent with the small differences in the overall hydrolysis rates as shown in Fig. 4a.

The PPC gels made at 90 and 120 °C (Fig. 6a and b) yield very similar chromatograms, but gels made at 140 °C (Fig. 6c) showed higher peaks for the first 60 min, which is represented for a larger area under the peak in the chromatogram. This is consistent with its higher overall rate of hydrolysis. After 180 min of gastric digestion, all chromatograms showed the same peaks and area, which shows that after 180 min, not just the protein hydrolysis is the same, but also the same fragments were formed.

The albumin from chicken egg white gels chromatograms showed minor differences between the protein gels made at 120 and 140 °C (Fig. 7b and c), while protein gels made at 90 °C (Fig. 7a) showed smaller peaks in the chromatograms. Indeed, the overall hydrolysis from these protein gels made at 90 °C was also much lower than the others (Fig. 4c).

The HPLC chromatograms of WPI gels made at 90 °C (Fig. 8a) are nearly identical to the chromatograms of gels made at 140 °C (Fig. 8c), and again this agreed with the protein hydrolysis values (Fig. 4d). Therefore, heating at 90 and 140 °C results in no significant differences in the hydrolysis rate and peptide profile. WPI gels made at 120 °C (Fig. 8b), however, showed smaller peaks between 0.1 and 5 kDa. As a lower hydrolysis (Fig. 5d) and higher hardness (Fig. 3) were found, we interpret this as this a more coherent gel, which disintegrated more slowly.

**Fig. 9:**
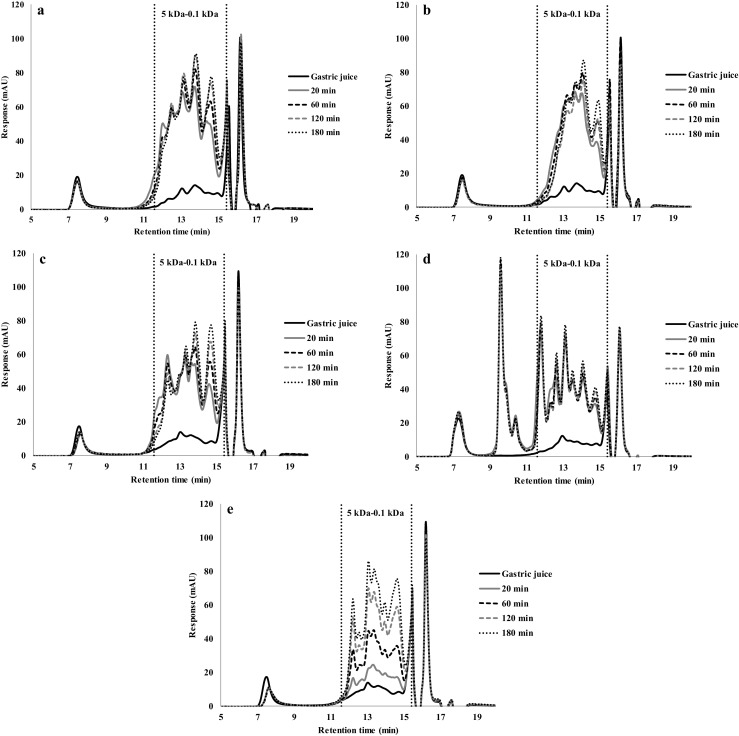
HPSEC profiles of gastric digestion of different protein sources in solution (5% protein) of: (**a**) Soy protein isolate (SPI), (**b**) Pea protein concentrate (PPC), (**c**) Albumin from chicken egg white; (**d**) Whey protein isolate (WPI) and (**e**) Casein

The HPSEC chromatograms of proteins in solution are shown in Fig. 9. SPI, PPC, albumin from chicken egg white and WPI sources, but not casein, showed fast hydrolysis during the first 20 min, which is also evident in the large number of peptides formed in the ranging from 5 to 0.1 kDa. The peptide peaks that are visible in the HPSEC chromatograms are overlapping with the peaks in Figs. 5, 6, 7, 8 and 9, indicating that the same peptides are cleaved off in gels and in solution. Also, larger peptide fragments are visible in the HPSEC chromatograms of proteins in solution. This is because all protein is present in solution, also large fragments. In the experiments with the gels, these large fragments most likely remained attached to the gel network.

The increase of the amount of smaller molecules (MW < 5 kDa) also was found by Chen [10]. They found as digestion time increased, larger molecules gradually shifted to smaller peptides as it was in this research. During proteolysis, the difference in the content of smaller peptide between samples gradually decreased. In SPI, β-conglycinin is more resistant to the proteolysis of pepsin than glycinin [40]. Therefore, the peptides formed during digestion correspond to glycinin hydrolysis [41].

The increased of smaller peptides during gastric digestion also was found by Laguna [42]. Reduced SDS-PAGE showed that during gastric digestion the molecules smaller than 15 kDa increased. This can be related to our results where a significant increase of peptides >5 kDa was found.

Luo [18] found that the peptide distribution for both albumin and WPI gels digested for 6 h showed that larger peptides (10–2 kDa) decreased steadily afterwards due to progressing hydrolysis, while the small peptides below 2 kDa increased throughout the whole process. An opposite result was found in our study, where peptides of different sizes (5–0.1 kDa) increased due to progressing protein hydrolysis for both gels and protein solutions.

The presence of a large number of intermediate products suggests that the peptic hydrolysis of dissolved denatured protein gels follow the “zipper-type” according to Linderstrøm-Lang’s theory [18].

## Conclusions

The rate of in vitro gastric plant protein hydrolysis was assessed as a function of their state (gel, solution) and history (gelation temperature). SPI and PPC in solution are both fast proteins: they were hydrolysed quickly in the first 20 min and then attach a plateau degree of hydrolysis. SPI gel, however, was hydrolysed very slowly, while PPC gel was hydrolysed quickly. This correlates well with the mechanical strength and porosity of the gels and the SEM studies of the gel morphologies. For comparison, whey protein gelled at 90 °C was hydrolysed slowly, but WPI gels heated at 120 or 140 °C were fast hydrolysers. Albumin gels were hydrolysed slowly irrespective of their gelling temperature but still showed somewhat faster hydrolysis with higher gelation temperatures. It is thus clear that by adapting the gel morphology, one can also adapt the gastric digestibility of food products based on protein gelation, and that plant-based proteins show a range of digestibility that is related to the properties of the gels.

## Electronic Supplementary Material

ESM 1 (DOCX 5506 kb)
