# Supplementary material for: The Effect of Gel Microstructure on Simulated Gastric Digestion of Protein Gels
Source: Food Biophys. 2018 Mar 5;13(2):124–38. doi: 10.1007/s11483-018-9518-7 (PMC5937947; doi:10.1007/s11483-018-9518-7)
Supplement: Supplementary file 1 — (DOCX 5506 kb) [file 11483_2018_9518_MOESM1_ESM.docx]

Excel

**Fig. 1** HPSEC profiles of protein solutions and protein gels made at different temperatures (90, 120 and 140 °C) of: **a** SPI (soy protein isolate), **b** PPC (pea protein concentrate), **c** Alb (albumin from chicken egg white) and **d** WPI (whey protein isolate)

**Fig. 2** Degree of hydrolysis of pea protein concentrate (PPC) and soy protein isolate (SPI) with and without NaCl added to the simulated gastric juice.
